# Endometriosis in a Man as a Rare Source of Abdominal Pain: A Case Report and Review of the Literature

**DOI:** 10.1155/2018/2083121

**Published:** 2018-01-31

**Authors:** Christina Rei, Thomas Williams, Michael Feloney

**Affiliations:** ^1^Creighton University School of Medicine, Omaha, NE, USA; ^2^Division of Urologic Surgery, Creighton University School of Medicine, Omaha, NE, USA

## Abstract

Endometriosis occurs when a tissue resembling endometrial glands and stroma grows in ectopic sites, commonly causing infertility and pain. This condition is most often seen in women of reproductive age, involving pelvic sites such as the ovaries, broad ligaments, uterosacral ligaments, and posterior cul-de-sac. Very rarely, endometriosis has also been found in the lower genitourinary tract of men. A 40-year-old man presented to his primary care physician with abdominal pain. Further imaging discovered a midline mass. Surgical removal of the mass and histological investigations led to the diagnosis of endometriosis. There are multiple theories on the etiology of both female and male endometriosis. The prevailing risk factor proposed in previous cases of male endometriosis is prolonged exposure to estrogen therapy. Should endometriosis become symptomatic, cessation of estrogen therapy and careful surgical intervention may successfully relieve the associated symptoms.

## 1. Introduction

Endometriosis has largely been studied in women, yet the precise etiology is unknown. In extremely rare cases, endometriosis is also found in men with a total of 16 cases previously reported in the literature [1–15]. In these cases, endometriosis was most commonly found attached to the bladder, lower abdominal wall, and inguinal region. It was previously hypothesized that either prolonged estrogen therapy [1, 3, 5, 7–9, 11, 13, 15], liver cirrhosis [2, 6], or chronic surgical inflammation [2, 6, 12] was a prerequisite for the development of endometriosis in males. We report a case of endometriosis in a 40-year-old man that was confirmed through immunohistochemical analysis. None of the commonly reported clinical risk factors for male endometriosis were evident in this patient; thus, we postulate hormonal alterations secondary to obesity as the main contributing factor to this patient's pathology.

## 2. Case Presentation

**Figure 1:**
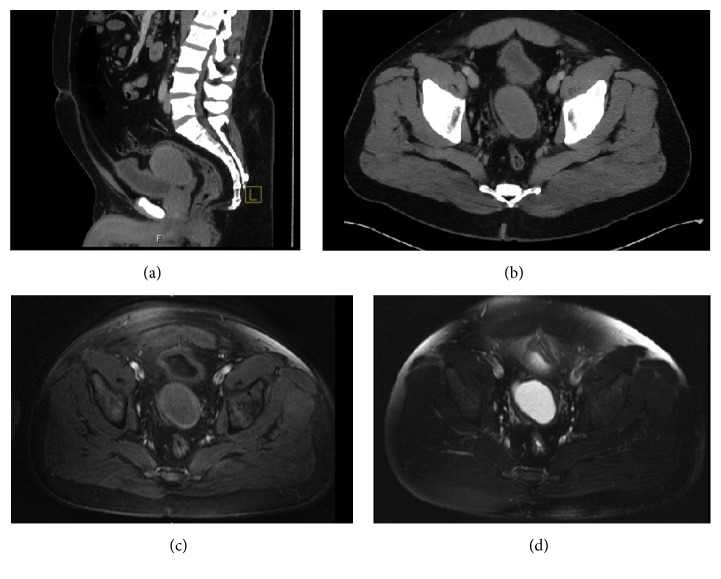
Computed tomography scan with contrast [(a) sagittal and (b) axial] displaying a complex midline cystic pelvic mass with thick walls found between the bladder and the rectum. Sagittal MRI of the pelvic mass with (c) T1-weighted imaging and (d) intensity on T2-weighted imaging.

A 40-year-old man with no significant past medical history presented to his primary care physician with worsening intermittent right lower quadrant abdominal pain radiating to his right flank. This pain was described as a constant dull ache with intermittent sharp pains. For the last three days, he had feelings of being bloated with progressive abdominal discomfort. His medical history was unremarkable aside from asthma, hypertension, and obesity with BMI of 35.7, while family history was significant for ovarian cancer in his mother. Of note, within the past week, he was treated with a course of high dose steroids for asthma exacerbation. His social history consisted of being a father to four biological children. Upon presentation, the patient denied dysuria, diarrhea, and blood or pain with bowel movements. On physical exam, he had a distended abdomen with right lower quadrant tenderness but no costovertebral angle tenderness, rebound tenderness, or guarding. A CT scan of the abdomen and pelvis revealed a large midline pelvic complex cystic lesion that appeared to arise from the right vas deferens (Figures 1(a) and 1(b)). Radiology recommended an MRI for clearer visualization and location of the mass based on results of the CT scan (Figures 1(c) and 1(d)). The MRI displayed intensity of the mass on T2-weighted imaging (Figure 1(c)). The distal portion of the right vas deferens was also dilated near the ejaculatory duct junction. The patient had followup appointments with general surgery and urology for surgical evaluation. A joint procedure between general surgery and urology was planned for cystourethroscopy, diagnostic laparoscopy, and excision of the pelvic mass.

A cystourethroscopy was performed to visualize the urethra and prostate. Diagnostic laparoscopy confirmed the presence of a mass anterior to the rectum and under the parietal peritoneum covering the dome of the bladder. The remainder of the procedure was converted to exploratory laparotomy for safer removal of the mass. The mass was palpated and found to be separate from the bladder and prostate and attached to the right vas deferens near the junction of the bladder and prostate. The right vas deferens was surgically divided proximal to the mass. A 9.0 × 5.6 × 5.3 cm, 125 g mass was successfully excised without evidence of invasion into surrounding structures or vasculature.

**Figure 2:**
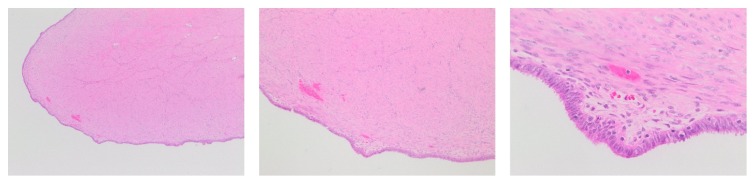
Immunohistochemical analysis staining with H&E at 100x, 200x, and 400x displaying epithelial cells and underlying stromal cells.

**Figure 3:**
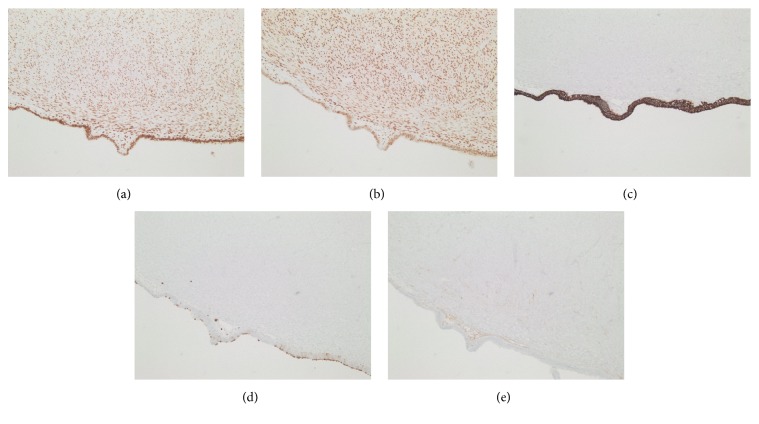
Immunohistochemical analysis stained (100x) (a) strongly positive for estrogen in epithelial and stromal cells, (b) strongly positive for progesterone receptor in epithelial and stromal cells, (c) strongly positive for CK7 in epithelial cells, (d) focally positive for CD15, and (e) positive for CD10 in the cytoplasm of stromal cells.

Gross exam of the mass in the operating room revealed a central cystic cavity with cloudy brown fluid. Further, the results from immunological stains performed by pathology revealed a highly unexpected diagnosis with an immunoprofile consistent with endometriosis. The H&E stain (Figure 2) displayed a layer of endometrial epithelium with underlying stroma. The cells lining the cystic mass stained strongly positive for CK7 (Figure 3(c)) and estrogen receptors (Figure 3(a)). CD10 stains were positive (Figure 3(e)) and CD15 staining was focally positive (Figure 3(d)) in the underlying stromal-like tissue. GATA-3 stain was negative. The patient was discharged on postoperative day two. In a two-week followup appointment, he had complete resolution of abdominal pain.

## 3. Discussion

Identifying the causative factors of endometriosis in men may shed light on the existing theories of endometriosis in women, which include retrograde transport, immunologic, induction, and coelomic metaplasia [16]. Further, this may provide evidence against the prevailing theory of retrograde transport as studied in female endometriosis. In the transport model, viable endometrial tissue is refluxed in a retrograde manner through the fallopian tubes during menstruation and grows on surrounding structures including the pelvis and peritoneum [16]. This theory would not explain the incidence of endometriosis in males who lack menstruation material. Thus, a more likely theory of induction of embryological remnants causing development of endometriosis should remain at the forefront.

A comprehensive review of risk factors, location, immunohistochemistry, and outcome of prior documented cases of endometriosis in males has been accomplished for comparison (Table 1). Most of the cases involve increased estrogen in men with liver cirrhosis [2, 6] or prostate cancer treated with long-term estrogen therapy [1, 3, 5, 7–9, 11, 13, 15]. Although this patient did not have the aforementioned risk factors, it is possible that his obesity with a BMI of 35.7 caused increased systemic estrogen levels. In the case reported by Zamecnik and Hostakova, the only identifiable risk factor was obesity as well [14]. Several studies have identified a clear, positive association between increased obesity in men and increased estrogen production [17]. This phenomenon is likely associated with increased aromatization activity of adipose tissue, overexpression of proinflammatory cytokines, insulin resistance, and hyperactivation of insulin-like growth factor pathways [17]. In relation to male endometriosis, it could be theorized that this increase in aromatization could provide pathologically elevated estrogen levels to drive the growth of endometriosis from remnant embryological cells in a male.

The induction theory of endometriosis hypothesizes that embryonic cell rests may persist in males and be induced into endometrial tissue. Divergence between male and female urogenital systems occurs from a common primordium, allowing for homologous structures to exist between the two genders [16]. The Müllerian ducts, which form the majority of the female genitourinary tract, normally disintegrate in males under the influence of anti-Müllerian hormone [9]. Thus, the cranially located appendix testes and caudally located prostatic utricle are typically the only vestigial structures derived from paramesonephric ducts [14]. The prostatic utricle serves as a homologue of the uterus and vagina [18]. It could therefore be theorized that while in the majority of males the Müllerian tissue atrophies completely, Müllerian cells may rarely persist between the ejaculatory duct and vas deferens when imperfect dissolution occurs [9]. These cell rests can further differentiate into endometrial tissue and lead to the development of endometriosis in males, likely under the influence of prolonged estrogen therapy or inflammation due to repeat surgeries [2, 6, 12]. The embryonic cell rest theory is the most congruent with the majority of cases of male endometriosis including the present case, as many of these lesions have occurred along the route of the Müllerian duct.

A third theory of endometriosis involves inadequate immune function. Various studies have cited alterations in both cell-mediated and humoral immunity [16] that coincide with the development of endometriosis. While this data shows promise, the exact mechanism needs to be further elucidated, especially in male patients, to show clear causation between the two.

Lastly, the coelomic epithelium metaplasia theory hypothesizes that, under the influence of certain signaling mechanisms, likely inflammatory cytokines, the peritoneal mesothelium undergoes metaplasia into tissue that resembles endometrial-like tissue and stroma. This theory could explain how women with Müllerian agenesis, who completely lack a uterus or have only a hypoplastic uterus, still show incidences of endometriosis [16]; however, it is less supportive than the induction theory. One case report of male endometriosis is in support of the coelomic epithelium metaplasia theory as the discovered endometriosis retained residual mesothelial phenotype, thus suggesting continuity and origin with a mesothelial cell layer [14].

The present and previously published cases of endometriosis in males may provide insight into the true origin of endometriosis. This presiding clinical evidence discredits the leading theory of retrograde menstruation as the dominant origin of endometriosis and points more towards an embryologic origin as the mechanism of this disease process.

## Figures and Tables

**Table 1:** Reported cases of endometriosis in males.

Source	Age	Risk factors	Clinical presentation	Immunohistochemistry	Location, size	Treatment	Followup
Beckman et al. [1]	78	Prolonged estrogen therapy	Not reported	Not reported	Prostatic urethral crest	Not reported	Not reported

González et al. [2]	52	Cirrhosis, spironolactone use, 2x inguinal hernia repair	Stabbing pelvic pain	Epithelium: ER+, PR+ Stroma: CD10+	R. inguinal area, attached to bladder serosa, 2.5 cm	Surgical resection	Not reported

Fukunaga [3]	69	9 years of hormonal therapy for prostatic adenocarcinoma, 1 year of radiotherapy and chemotherapy	Swelling of the left testis on a routine examination	Vimentin+, CD10+, ER+, PR+	L. paratestis, 5.2 × 3.1 × 3.0 cm	Bilateral orchiectomy	Not reported

Giannarini et al. [4]	27	Not reported	2 weeks of postcoital left scrotal pain	ER+, PR+, CK7, 8, 18, 19+, vimentin, CEA, CD10−	Head of the L. epididymis, 1.7 cm	Surgical resection	Asymptomatic at 5 years

Young and Scully [5]	82	3 years of DES for prostatic adenocarcinoma	Palpable firm mass on the tail of the epididymis on routine examination	Not reported	Between vas deferens and testis, close to the tail of the epididymis, 5 cm	Bilateral orchiectomy	Died 9 months later due to metastatic prostatic adenocarcinoma

Jabr and Mani [6]	52	Cirrhosis secondary to Hep. C; inguinal hernia repair with mesh	Right lower quadrant pain	ER+, PR+, CD10+	Cystic mass attached to urinary bladder and right inguinal area, 4.5 × 2.5 cm	Surgical resection	Asymptomatic

Martin and Hauck [7]	83	TACE therapy for prostatic adenocarcinoma	Not reported	Not reported	Lower abdominal wall	Not reported	Not reported

Oliker and Harris [8]	80	Prolonged hormonal therapy	Not reported	Not reported	Bladder	Not reported	Not reported

Pinkert et al. [9]	50	TACE therapy for prostatic adenocarcinoma	Hematuria, hydroureter	H&E	Ulceration surrounding trigonal area, bladder muscular wall	Surgical resection, discontinued hormonal therapy	Asymptomatic at 4 years

Tulunay et al. [10]	43	Within clear cell carcinoma of tunica vaginalis	Hemoptysis, abdominal pain, weight loss	H&E	Left paratestis	Left orchiectomy	Died 2 weeks later due to tumor progression

Schrodt et al. [11]	73	5-year hormonal therapy for prostate adenocarcinoma	Right hydronephrosis	Not reported	Right ureterovesical junction	Not reported	Not reported

Simsek et al. [12]	49	Inguinal hernia repair ×3	Intraoperative hernia repair, mass discovered along the spermatic cord	H&E	Left ductus deferens, 8 × 7 × 6 cm	Surgical resection	Not reported

Taguchi et al. [13]	74	Radical prostatectomy for prostatic adenocarcinoma; leuprorelin and ethinylestradiol for 5 years	Painless macrohematuria	ER+, PR+, CD10+, PSA−	Left ureteral orifice, 3 cm	Surgical resection, discontinued hormonal therapy	Tumor shrank on imaging; no PSA elevation at 6 months

Zamecnik and Hostakova [14]	46	Obesity, BMI of 31	Cyst found adjacent to seminoma	Epithelium: ER+, PR+, CK5,6,7+, calretinin+, EMA+ Stroma: PR+, calretinin+, CD10+	Within mesothelial cyst of tunica vaginalis; 4 mm focus of endometriosis found in 7 mm cyst	Right-sided orchiectomy	Not reported

Scully [15]	Not reported	Hormonal therapy for prostate adenocarcinoma	Not reported	Not reported	Scrotum	Not reported	Not reported

Scully [15]	Not reported	Hormonal therapy for prostate adenocarcinoma	Not reported	Not reported	Scrotum	Not reported	Not reported

Present case	40	Obesity, BMI of 35.7	Right lower quadrant abdominal pain radiating to the right flank	CK7+, ER+, CD10+, CD15+, GATA-3−	Right vas deferens, 9.0 × 5.6 × 5.3 cm	Surgical resection	Asymptomatic at 2 weeks
